# Food Toxicology and Food Safety: Report of the 3rd International Electronic Conference on Foods: Food, Microbiome, and Health—A Celebration of the 10th Anniversary of Foods’ Impact on Our Wellbeing

**DOI:** 10.3390/foods11244099

**Published:** 2022-12-19

**Authors:** Dirk W. Lachenmeier, Paula A. Oliveira, Agata Urszula Fabiszewska, Cristina Maria Dias Soares, Jong H. Kim

**Affiliations:** 1 Chemisches und Veterinäruntersuchungsamt (CVUA) Karlsruhe, Weissenburger Strasse 3, 76187 Karlsruhe, Germany; 2 Department of Veterinary Sciences, University of Trás-os-Montes and Alto Douro (UTAD), 5001-801 Vila Real, Portugal; 3 Clinical Academic Center of Trás-os-Montes and Alto Douro, University of Trás-os-Montes and Alto Douro (UTAD), 5001-801 Vila Real, Portugal; 4 Inov4Agro, Center for the Research and Technology of Agro-Environmental and Biological Sciences (CITAB), University of Trás-os-Montes and Alto Douro (UTAD), 5001-801 Vila Real, Portugal; 5 Institute of Food Sciences, Warsaw University of Life Sciences, 159c Nowoursynowska St., 02-776 Warsaw, Poland; 6 REQUIMTE/LAQV, Instituto Superior de Engenharia do Instituto Politécnico do Porto, Rua Dr. António Bernardino de Almeida, 431, 4200-072 Porto, Portugal; 7 Foodborne Toxin Detection and Prevention Research Unit, Western Regional Research Center, Agricultural Research Service, United States Department of Agriculture, 800 Buchanan St., Albany, CA 94710, USA

**Keywords:** food toxicology, food safety, novel food, mammary carcinogenesis, *Yarrowia lipolytica*, microbial oil, benzaldehyde, drug repurposing, cannabidiol

## Abstract

The purpose of the conference session summarized in this article was to bring together international experts on food toxicology and food safety and share the current scientific knowledge on these topics. The presentations covered a wide range of interdisciplinary issues, including (i) the impact of diet on body weight and health outcomes including results from animal models of carcinogenesis, (ii) methods for microbial oil extraction, (iii) food processing and its impact on food safety and health, (iv) novel compounds to avoid mycotoxin contamination of agricultural products, and (v) the safety of cannabidiol in food supplements based on *Cannabis sativa* extracts. Some of the conclusions of the presentations included that correct food choices may impact on the risk of non-communicable diseases such as cancer, that food processing may have an influence on health, by either reducing or increasing risks, and that research regarding novel compounds is important, which may have preventive but also detrimental effects on health.

## 1. Introduction

**Figure 1:**
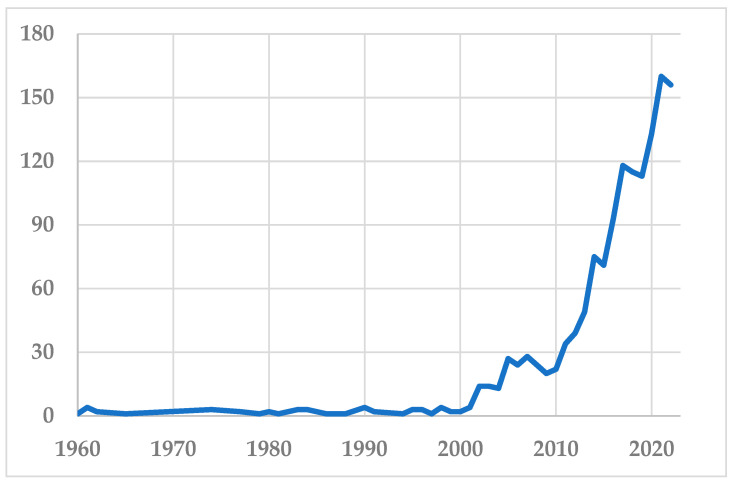
Scientific publications 1960–2022 on food toxicology. Source: PubMed, National Library of Medicine, Bethesda, MD, USA. Search term: “food toxicology”, search conducted 16 December 2022.

In recent decades, the importance of food toxicology (Figure 1) and food safety in general has grown enormously. The desire to be able to consume food that is not detrimental to our wellbeing in a healthy environment has increased the public’s awareness of food toxicology issues. Food toxicology in the broadest sense is the connection between components of foods and possible health risks. Various techniques may be applied including experimental in vitro and in vivo studies, exposure assessments, epidemiological studies, and advanced mathematical models for risk assessment. Ultimately, the aim of food toxicology is to provide a scientific basis to ensure food safety. In the subfield of regulatory toxicology, the knowledge of food toxicology may be used as a scientific basis for food policy decisions, e.g., about maximum limits of certain compounds in foods, as well as a basis for enforcement action by authorities, i.e., taking unsafe foods from the market. Consumers also consider food safety to be one of the most important topics [1].

Food Toxicology was added in 2021 as a section of the MDPI Foods journal. Following the success of last year’s conference [2], during this session of the Foods 2022 conference, the organizers wanted to take the chance to introduce this section. For that, international experts in food toxicology and food safety were invited to provide an update on current hot topics in this field.

**Figure 2:**
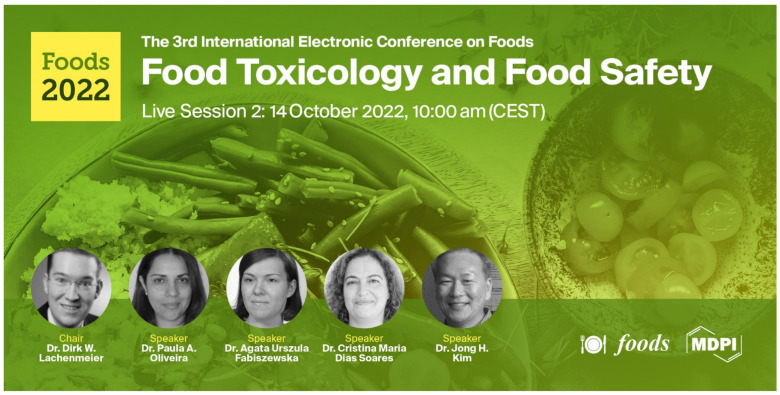
Speakers of the Foods 2022 Conference live session 2 on food toxicology and food safety.

This article summarizes the main outcomes of the second live session of the 3rd International Electronic Conference on Foods: Food, Microbiome, and Health—A Celebration of the 10th Anniversary of Foods’ Impact on Our Wellbeing entitled “Food Toxicology and Food Safety”. Dirk W. Lachenmeier (CVUA Karlsruhe, Karlsruhe, Germany) chaired the conference session and was also a speaker. The participating experts were Paula A. Oliveira (UTAD, Vila Real, Portugal), Agata Urszula Fabiszewska (Warsaw University, Warsaw, Poland), Cristina Maria Dias Soares (REQUIMTE/LAQV, Porto, Portugal), and Jong H. Kim (USDA, Albany, CA, USA) (Figure 2). The audience of more than 50 participants contributed with questions and comments. The conference session was live streamed on MDPI’s YouTube channel, and a video recording is available on the conference webpage (https://foods2022.sciforum.net/#live_session_recordings (accessed on 27 October 2022)). The conference evaluation committee chose Agata Urszula Fabiszewska (Institute of Food Sciences, Warsaw University of Life Sciences) for the Best Speaker Award.

This article provides a synopsis of the live session, held on 14 October 2022, providing summaries from all speakers.

## 2. Summary of Conference Presentations

### 2.1. Paula A. Oliveira: Impact of Western Diet on Body Weight and Food and Water Consumption: Data from a Rat Model of Mammary Carcinogenesis

Western diet (WD) is characterized by a high daily intake of processed carbohydrates, saturated fats, and salt, appearing to be the blame for several illnesses, such as cancer, atherosclerosis, dementia, and metabolic disorders. Mammary cancer victimized approximately 685,000 people in the year 2020. These data reinforce the idea that much remains to be done in mammary cancer research concerning the diet influence on its development.

This work aimed to evaluate the effects of two different diets on body weight and food and water consumption in a rat model of chemically induced mammary cancer.

Twenty-eight female rats were divided into four groups (n = 7): WD; WD + *N*-methyl-*N*-nitrosourea (MNU); standard diet (SD); and SD + MNU. WD + MNU and SD + MNU animals received an intraperitoneal injection of the carcinogen MNU, at seven weeks of age. The animals were supplied with water and food ad libitum. The WD groups received a WD with 60% of total calories coming from fat, while the SD groups received a standard maintenance laboratory diet. Body weight and food and water consumption were recorded every week for 20 weeks. This experimental work was approved by the institutional ethics committee (834-e-CITAB-2020).

A gradual increase in the animals’ body weight was observed throughout the study, which is associated with their normal growth. Accordingly, food and water intake increased gradually over the study. No differences in body weight or food or water consumption were observed between the groups (*p* > 0.05). A significant correlation was observed between food and water consumption (*p* < 0.01).

Diet or MNU administration seemed to not significantly influence the feeding pattern or animals’ body weight in the rat model of chemically induced mammary cancer.

### 2.2. Agata Urszula Fabiszewska: Non-Conventional Methods of Oleaginous Yeast Pretreatment for Microbial Oil Extraction

Lipids (microbial oil) are accumulated in the cells of oleaginous microorganisms, including yeast, in amounts exceeding 20% of dry mass, which are a valuable source of fatty acids in the human diet. To facilitate the extraction of storage lipids from cells, methods of physical and chemical pretreatment of biomass are used to break the barrier of the cell wall and membrane of these microorganisms to the action of organic solvents, which are used during traditional extraction. The purpose of the study was to evaluate the effectiveness of unconventional methods of extracting microbial oil from *Yarrowia lipolytica* yeast cells. Pulsed electric field (PEF), cell disintegration by ultrasonic waves, and high-pressure homogenization (HPH) were used. The use of unconventional methods turned out to be ineffective in the extraction of intracellular lipids of the yeast compared to methods involving organic solvents such as chloroform, methanol, and hexane. Nevertheless, the use of a pulsed electric field with a field strength of 200 J/g or high-pressure homogenization (700 and 1100 bar) proved to be effective as pre-treatment techniques of *Y. lipolytica* yeast cells (cell permeabilization) for the high yield extraction of intracellular lipids using the extraction method with organic solvents [3].

### 2.3. Cristina Maria Dias Soares: Food Processing and Its Potential Impact on Food Safety and Health

The increasing complexity of production systems for food influences consumers’ concerns about its quality and safety. Traditionally, the most important reasons for foods to be processed are to make them last the longest without deteriorating and to improve their organoleptic characteristics. Processing is also often intended to add nutritional value, improve the final appearance, facilitate consumption, and reduce the time of homemade food preparation. Currently, food processing and preservation technologies include, among others, the use of heat or heat exchange, preservation by cold, evaporation or dehydration, the addition of chemicals, and fermentation processes [4]. Despite all the advantages discussed, processing food at high temperatures may also lead to the loss of nutritionally essential constituents. Additionally, toxic compounds may be formed (i) from the interaction between compounds in food with each other, (ii) between these and exogenous constituents, such as additives or the reaction medium itself, and (iii) between substances resulting from the initial interactions. One of these compounds is acrylamide, formed through the Maillard reaction in fried, baked, and roasted foods rich in reducing sugars and asparagine [5]. Besides the formation of toxic compounds, thermal processing can also be used to increase the nutritional value of undervalued foods, such as acorns, to prepare functional foods with improved organoleptic properties and increased antioxidant activity with the formation of melanoidins and low advanced glycation end products. Moreover, thermal processing can increase the safety of consuming foods such as seaweeds, which contain high amounts of iodine and arsenic. After boiling, leaching of these elements can increase the safety of this food [6]. In conclusion, it is crucial to assess the drawbacks and benefits of food processing considering its health relevance while balancing the associated health risks and benefits for consumers.

### 2.4. Jong H. Kim: Benzaldehyde Use to Protect Seeds from Foodborne Fungal Pathogens

Contamination of food/crops by fungi is a recurring food safety and security issue. There is limited efficacy with conventional seed sanitation methods, directly affecting food safety. For instance, the insufficient elimination of mycotoxin-producing fungi contaminating seed surfaces can result in high mycotoxin contamination. In 2020, peanut seeds in southeast United States exhibited a significantly low germination rate during crop season, which was preceded by a high frequency of aflatoxin (AF) contamination in 2019 [7]. The prevalence of the *Aspergillus flavus* fungicide-resistant strains was the root cause of the high AF contamination and poor seed quality. Heat treatment (pasteurization/blanching) is one of the strategies that can inactivate microbial contaminants in agricultural/food production. However, excessive heat treatment can negatively affect the quality of the crops/food products. Hurdle technology is an approach where the coapplication of different types of preservation methods at reduced individual intensities can increase the effectiveness of antimicrobial treatments. In this study, a new seed sanitation formula was investigated by examining Generally Regarded As Safe (GRAS) molecules such as natural compounds/structural derivatives or redox-active molecules repurposed from FDA-approved food additives as active ingredients. Selected benzo derivatives, previously shown to inhibit mycotoxin production, could function as heat-sensitizing agents, enhancing the efficacy of sanitation against fungi. When benzo derivatives and mild heat (57.5 °C) were co-administered for 90 s (in vitro), the co-application achieved >99.999% microbial elimination, while the independent application of either agent alone allowed pathogen survival. For seed treatments, the coapplication of a benzo derivative (3 mM) and mild heat (50 °C, 20 to 30 min) completely inhibited the germination of aflatoxin-producing *A. flavus* contaminated on *Brassica rapa* Pekinensis (Chinese cabbage hybrid) seeds, while seed germination rate was not affected. In summary, benzo-derivative-based heat sensitization could be a promising tool to achieve safe and cost-effective pathogen control in agriculture/food production [8].

### 2.5. Dirk W. Lachenmeier: Does Cannabidiol (CBD) in Food Supplements Pose a Serious Health Risk? Consequences of the EFSA Clock Stop Regarding Novel Food Authorization

At present, foods containing cannabidiol (CBD) and other cannabinoids are internationally being widely advertised and sold in increasing quantities [9]. In the European Union (EU), these products require pre-marketing authorization under the novel food regulation, so all available CBD oils and CBD-containing food supplements in the EU are currently placed on the market with an infringement of food laws [10]. Currently, 19 CBD applications are being evaluated by the European Food Safety Authority (EFSA) [11]. During the initial assessment of the application files, EFSA found several knowledge gaps that need to be addressed before the safety evaluation of CBD can be concluded. That is, the effect of CBD on the liver, gastrointestinal tract, endocrine system, nervous system, psychological function, and reproductive system must be clarified [11]. Nevertheless, the available literature allows a benchmark dose (BMD)–response modeling of several bioassays, resulting in a BMD lower confidence limit (BMDL) of 20 mg/kg bw/day for liver toxicity in rats [12]. Human data in healthy volunteers found increases in the liver enzymes alanine aminotransferase (ALT) and aspartate aminotransferase (AST) in a study at 4.3 mg/kg bw/day, which was defined by EFSA as a lowest observed adverse effect level (LOAEL) [13]. The EFSA panel currently concluded that the safety of CBD as a novel food cannot be evaluated, leading to a so-called clock stop of applications until the applicants provide the required data [11]. Meanwhile, it is suggested that CBD products still available on the EU market despite the lack of authorization must be considered “unsafe” [12]. Products exceeding a reference dose of 10 mg/day must be considered “unfit for consumption” (Article 14(1) and (2) (b) of Regulation No. 178/2002), while those exceeding the human LOAEL must be considered “injurious to health” (Article 14(1) and (2) (a) of Regulation No. 178/2002 [14]).

## 3. Conclusions

As this conference session has confirmed, the study of food safety and toxicology has an important impact on improving health and wellbeing, which was the overarching topic of the Foods 2022 conference. The ultimate goal of food toxicology to provide safe foods is still as important today as it was 60 years ago in the article of Sebrell, which is the first indexation of food toxicology in PubMed [15].

Correct food choices may impact on the risk of noncommunicable diseases such as cancer, either directly or by the link between obesity and cancer risk [16]. Additionally, food processing may have an influence on health, by either excluding or reducing risks (e.g., microbial risk by heating or sterilization, or chemical risks by reducing compounds such as iodine); on the other hand, processing may also increase risks by the formation of compounds with adverse health effects such as furan or acrylamide. Finally, the research interests regarding novel compounds that may be used in the food context were pointed out, e.g., benzaldehyde as a compound to prevent mycotoxin production or cannabidiol as a compound widely used in food supplements.

The authors hope that this conference has stimulated interest and future research on food toxicology and food safety. Hopefully, another conference dedicated to these topics will be possible in the near future as an in-person meeting, when the COVID-19 pandemic has been overcome.

## Data Availability

No new data were collected or analyzed in this study. Data sharing is not applicable to this article.
